# Pigmentation-dependent skin injury thresholds with nanosecond single-pulse laser exposure

**DOI:** 10.1117/1.JBO.31.8.085001

**Published:** 2026-08-08

**Authors:** Junior Arroyo, Jiaxin Zhang, Madhavi Tripathi, Junhao Zhang, Nethra Venkatayogi, Jamie Enslein, Muyinatu A. Lediju Bell

**Affiliations:** a Johns Hopkins University, Department of Biomedical Engineering, Baltimore, Maryland, United States; b Johns Hopkins University, Department of Electrical and Computer Engineering, Baltimore, Maryland, United States; c Johns Hopkins University, Department of Computer Science, Baltimore, Maryland, United States

**Keywords:** laser safety, skin injury threshold, pigmentation, laser-tissue interaction, individual typology angle

## Abstract

**Significance:**

Maximum permissible exposure limits defined in ANSI Z136.1 are broadly protective, but they do not explicitly account for pigmentation differences in skin. As a result, the lack of experimental data quantifying the effect of pigmentation on injury thresholds constrains accurate interpretation when melanin is a primary optical absorber, which has direct implications for the performance of optics-based imaging methods.

**Aim:**

We experimentally determined the pigmentation-dependent laser exposure dose that produces a visible injury in 50% of exposed sites (i.e., ${\mathrm{ED}}_{50}$ injury thresholds) with nanosecond, 750-nm-wavelength pulses, and we provide the first known quantification of relationships between melanin index (MI) and individual typology angle (ITA) or erythema index (EI) through colorimetric measurements.

**Approach:**

Fifty total abdominal skin sites per swine were identified on three swine with different pigmentation phenotypes (median ITA values per swine ranged −18° to 87°). Single-laser pulses with 5-ns pulse duration, 750-nm-wavelength, and energies of 35, 40, 50, 60, and 65 mJ per pulse were delivered to 10 sites per energy level per swine. Laser-exposed sites were visually assessed at early post-exposure intervals, followed by colorimeter measurements of MI, ITA, and EI. Probit regression analysis was used to compute the ${\mathrm{ED}}_{50}$ thresholds, followed by a polynomial regression analysis conducted to relate ITA to MI and EI to MI.

**Results:**

Dark-skinned swine (median ITA=−18°) exhibited a 27.7% lower ${\mathrm{ED}}_{50}$ ($323.94\;\mathrm{mJ}/{\mathrm{cm}}^{2}$) than light-skinned swine (median ITA=69.5°, ${\mathrm{ED}}_{50}=448.36\;\mathrm{mJ}/{\mathrm{cm}}^{2}$), which is consistent with increased melanin-mediated absorption in the darker-skinned swine. The lightest-skin swine (median ITA=87°) did not develop visible erythema within the tested exposure range, preventing ${\mathrm{ED}}_{50}$ estimation. Mathematical MI–ITA and MI–EI relationships revealed pigmentation-dependent optical and inflammatory responses across the three swine.

**Conclusions:**

The measured differences in ${\mathrm{ED}}_{50}$ values quantify the impact of skin pigmentation on laser-induced injury risk and demonstrate that darker skin has greater susceptibility to damage with the investigated laser parameters, relative to lighter skin. This foundational work addresses a previously uncharacterized source of biological variability that is relevant for the interpretation of laser safety margins and the design and evaluation of optics-based systems.

## 1 Introduction

The safe application of pulsed laser technology in optics-based imaging modalities depends on accurate knowledge of exposure levels that prevent skin injury. ANSI Z136.1^1^ provides exposure limits to prevent adverse biological effects on the skin, referred to as the maximum permissible exposure (MPE), which varies as a function of laser parameters (e.g., pulse width, wavelength). These limits were derived from experimentally determined ${\mathrm{ED}}_{50}$ thresholds, defined as the radiant exposure (i.e., energy fluence) that produces a visible injury in 50% of exposed sites, scaled by conservative safety margins derived primarily from animal skin models and limited human studies.^2^ Although the ${\mathrm{ED}}_{50}$ datasets used to derive MPE values are distributed across multiple studies and technical reports rather than consolidated into a single source, it is well-established that ${\mathrm{ED}}_{50}$ thresholds are intrinsically dependent on pulse duration, wavelength, and the optical and thermal properties of tissue.^3^^–^^5^

The MPE limits are intended to be broadly protective and are therefore applied uniformly across diverse skin tones, without explicitly accounting for pigmentation-dependent differences in optical and thermal properties. Melanin significantly determines how light propagates through the skin by strongly absorbing visible wavelengths (and near-infrared wavelengths to some extent), whereas melanosomes introduce scattering that further modifies the local fluence distribution and heat deposition rate.^6^ These melanin-mediated effects are expected to introduce significant variability in ${\mathrm{ED}}_{50}$ thresholds across skin tones, as previously suggested by Rockwell et al.,^7^ yet current safety limits do not incorporate quantitative estimates of this variability.

In this context, pigmentation-dependent differences in optical energy deposition can influence the performance of optics-based devices across skin tones, with implications for clinical applicability in diverse patient populations.^8^^–^^11^ This issue is particularly relevant for modalities such as photoacoustic imaging, where signal amplitude scales with absorbed optical energy.^12^ In particular, a specific percent increase in the optical absorption coefficient is expected to yield the same percent increase in the photoacoustic signal amplitude.^13^ These improvements are most directly reflected in signal-related image quality metrics such as target detectability and signal-to-noise ratio, whereas spatial resolution is mainly governed by acoustic detection bandwidth, element size, and detection aperture.^14^^,^^15^ Signal amplitude, image quality, and spatial resolution together influence diagnostic performance, particularly when target visibility is limited by optical attenuation.

However, in translational photoacoustic systems, increasing pulse energy is constrained by ANSI Z136.1 skin MPE limits when optical energy is delivered through or onto skin, thereby capping the usable laser pulse energy. Previous Monte Carlo–based modeling studies of light transport in skin have demonstrated that melanin concentration and pigmentation phenotype alter optical fluence distributions and induce spectral coloring.^9^ As a result, exposure levels selected within established safety guidelines may correspond to different margins relative to injury thresholds across pigmentation phenotypes, impacting achievable signal strength, light penetration depth, and diagnostic performance. Quantifying pigmentation-dependent injury thresholds therefore provides essential experimental context for interpreting laser safety margins and optimizing system performance while remaining compliant with existing laser safety guidelines.

Objective characterization of skin pigmentation is important to the evaluation of pigmentation-dependent laser safety. Optical energy deposition is influenced by melanin-associated optical absorption, which is not accurately captured by categorical skin-type labels. For example, the Fitzpatrick scale, although widely used in dermatology, was originally developed to describe self-reported sun sensitivity and tanning response, rather than to directly quantify continuous variation in pigmentation.^16^ By contrast, individual typology angle (ITA), erythema index (EI), and melanin index (MI) provide objective, continuous, instrument-based measurements of skin color and pigmentation. ITA is calculated from CIE L*a*b* color space using the L* lightness and b* yellow-blue components of colorimeter measurements and is commonly used as an objective skin typology classification for photobiological applications.^17^ The original ITA formulation used L* and b* because these components showed the strongest correspondence with the visually assessed intensity of constitutive pigmentation. By contrast, the a* red-green component was influenced by both melanic pigmentation and natural skin vascularity, reducing its specificity for the classification of constitutive pigmentation.^17^ EI and MI are colorimeter-based indices associated with hemoglobin-related redness and melanin-associated pigmentation, respectively, and are commonly used to quantify erythema and pigmentation, respectively, in dermatology applications.^18^ Therefore, these three metrics (i.e., ITA, EI, and MI) are better suited for the analysis of laser-tissue interactions, relative to more subjective approaches, such as the Fitzpatrick scale.

The study herein presents experimentally determined pigmentation-dependent ${\mathrm{ED}}_{50}$ values for nanosecond, 750-nm-wavelength pulses in a swine model with different pigmentation phenotypes (characterized with colorimeter measurements), with two primary contributions. First, we report the ${\mathrm{ED}}_{50}$ thresholds across a range of swine skin pigmentation phenotypes using ITA, EI, and MI to characterize the evaluated regions, addressing a gap in prior studies that classified skin using coarse categorical labels. Second, we provide a mathematical expression that characterizes the temporal evolution of MI–ITA and MI–EI relationships across the full range of possible ITA values (i.e., between −90° and 90°) and multiple MI values. These contributions establish a quantitative experimental basis for characterizing and modeling pigmentation-dependent variability in laser-induced injury thresholds, providing critical context for interpreting existing laser safety guidelines.

## 2 Methods and Materials

### 2.1 Experimental Design and Laser Exposure

The Institutional Care and Use Committee of Johns Hopkins University, an AAALAC-accredited institution, approved the protocol to conduct the *in vivo* studies described herein. Animals and procedures were in compliance with the US Public Health Service’s Policy on Humane Care and Use of Laboratory Animals,^19^ the US Department of Agriculture’s Animal Welfare Act,^20^ and the National Research Council’s Guide for the Care and Use of Laboratory Animals.^21^

Experiments were performed on three female Yucatan miniature pigs (*Sus scrofa domesticus*) purchased from Sinclair BioResources (Columbia, Missouri, United States), with body weights ranging from 26 to 31 kg and varying degrees of skin pigmentation. This swine breed was previously used to quantitatively assess melanin distributions in swine skin^22^ and was selected for our study because of the associated cutaneous pigmentation, which is absent in nonpigmented breeds (e.g., Yorkshire swine).^23^ In particular, Yucatan skin pigmentation is suitable for laser-skin-injury threshold studies at melanin-absorbing wavelengths (e.g., 750 nm).^23^

Based on established ITA classification criteria, the local skin tones of the swine in our study were classified as very light (ITA>55°), light (41°<ITA≤55°), intermediate (28°<ITA≤41°), tan (10°<ITA≤28°), brown (−30°<ITA≤10°), or dark (ITA≤−30°).^17^ Based on these classifications, the swine herein are referred to as D1 (i.e, dark-skinned swine with abdominal ITA values ranging from −79° to 8° prior to laser delivery), L1 (i.e., light-skinned swine with ITA values ranging from 57° to 82°), and L2 (i.e., light-skinned swine with ITA values ranging from 63° to 90°). The ${\mathrm{ED}}_{50}$ injury threshold analysis was performed using data from swine D1 and L1, considering that no erythema (i.e., reddening characteristic of tissue damage) was detected in swine L2, which prevented regression analyses. The derivation of the mathematical expressions relating colorimeter-based optical measurements (i.e., MI–ITA and MI–EI) was performed using data from swine D1, L1, and L2.

**Fig. 1:**
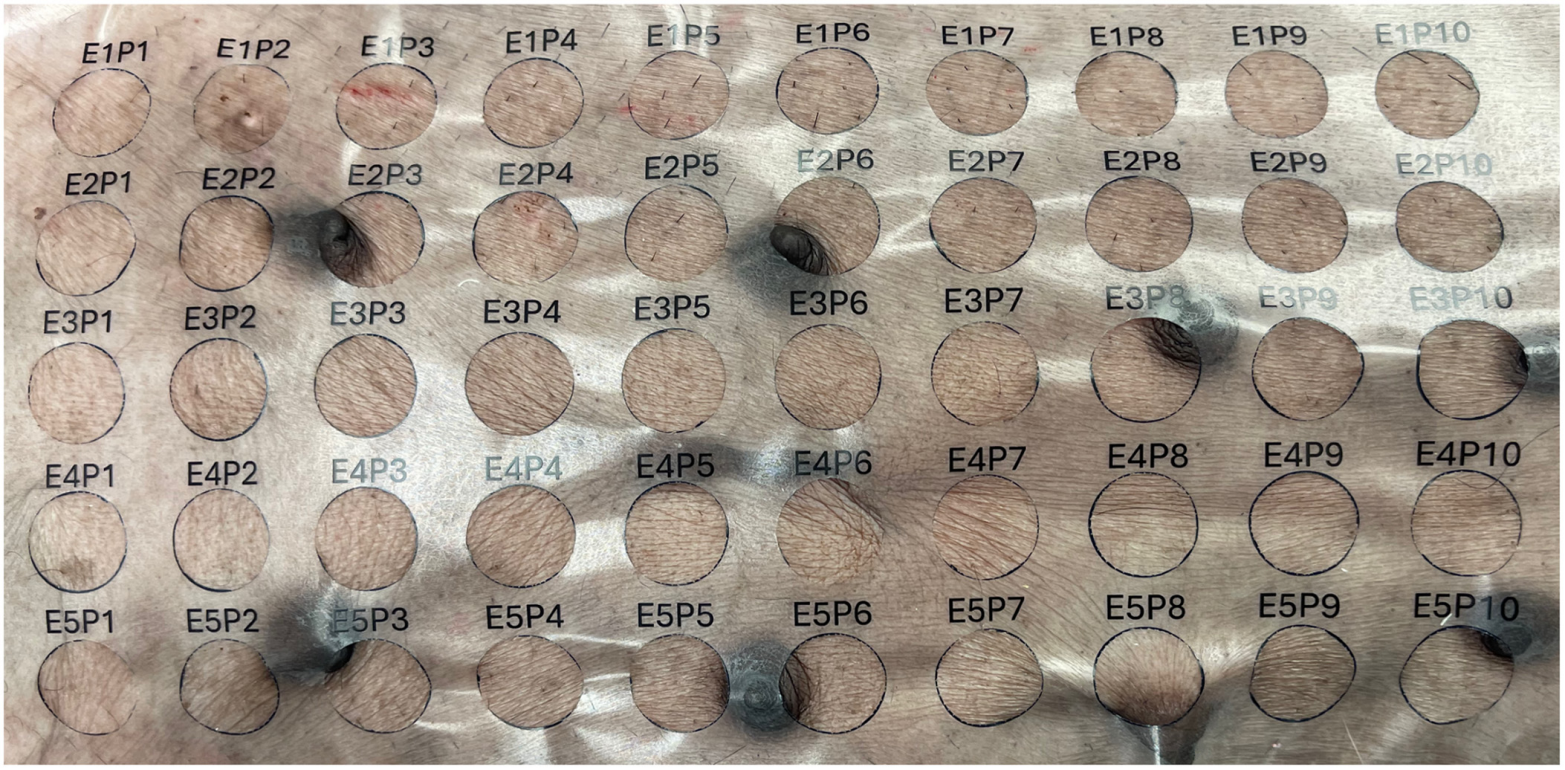
Sticker grid placed on the shaved abdominal skin.

The ventral abdominal skin was shaved and cleaned to remove surface hair, minimize hair-related scattering above the skin surface, and improve optical coupling before laser exposure. No visible erythema was observed after shaving and before laser exposure. A rectangular grid sticker was placed on the shaved abdominal skin to delineate the regions designated for laser exposure, as shown in Fig. 1. The entire sticker grid measured 157  mm×310  mm and contained 50 20-mm-diameter circular regions, arranged in 5 rows and 10 columns. Each designated site was uniquely identified by its assigned pulse energy level (E1 to E5) and site number (P1 to P10). The swine were positioned under a warming pad to maintain normothermia throughout the procedure.

Laser energy was delivered to the designated sites using a 5-mm-diameter optical fiber with a numerical aperture of 0.22 connected to a Phocus Mobile laser (Opotek, Carlsbad, California, United States) operating at a wavelength of 750 nm. Before laser light delivery, the fiber was manually positioned normal to the skin surface and held in gentle contact with each designated exposure site for several seconds to ensure optical coupling. Single pulses of 35, 40, 50, 60, and 65 mJ energy (equivalent to 178.25, 203.72, 254.65, 305.58, and $331.04\;\mathrm{mJ}/{\mathrm{cm}}^{2}$ radiant exposure, respectively) with a pulse duration of 5 ns were applied to each site. To monitor and compensate for known fluctuations and maintain pulse-to-pulse variations within ±2  mJ of the assigned energy level E1 to E5, the internal power meter of the laser and a custom command-line interface were employed, using our previously described energy calibration and monitoring methods,^24^^–^^26^ with measurements for a single pulse used to adjust energy (if necessary) prior to delivering the next pulse.

### 2.2 Skin Tone Classification

Similar to previous studies in dermatological and biomedical applications,^27^^,^^28^ skin tone in each designated region was quantified by measuring ITA using a calibrated SkinColorCatch colorimeter (Delfin Technologies Ltd, Kuopio, Finland). This measurement was obtained 20 min after abdominal skin shaving to minimize measurements on any irritated surfaces. For each measurement, the SkinColorCatch device reported CIE L*a*b* color values, ITA, MI, and EI. ITA was calculated from the L* and b* values using the formulation proposed by Chardon et al.^17^ as:

$$\mathrm{ITA}=\arctan(\frac{L^{*}-50}{b^{*}})\times \frac{180}{\pi },$$ (1)

where L* represents lightness and b* represents the yellow-blue color axis. EI and MI were obtained directly from the colorimeter as instrument-reported indices associated with erythema-related redness and melanin-associated pigmentation, respectively.

**Fig. 2:**
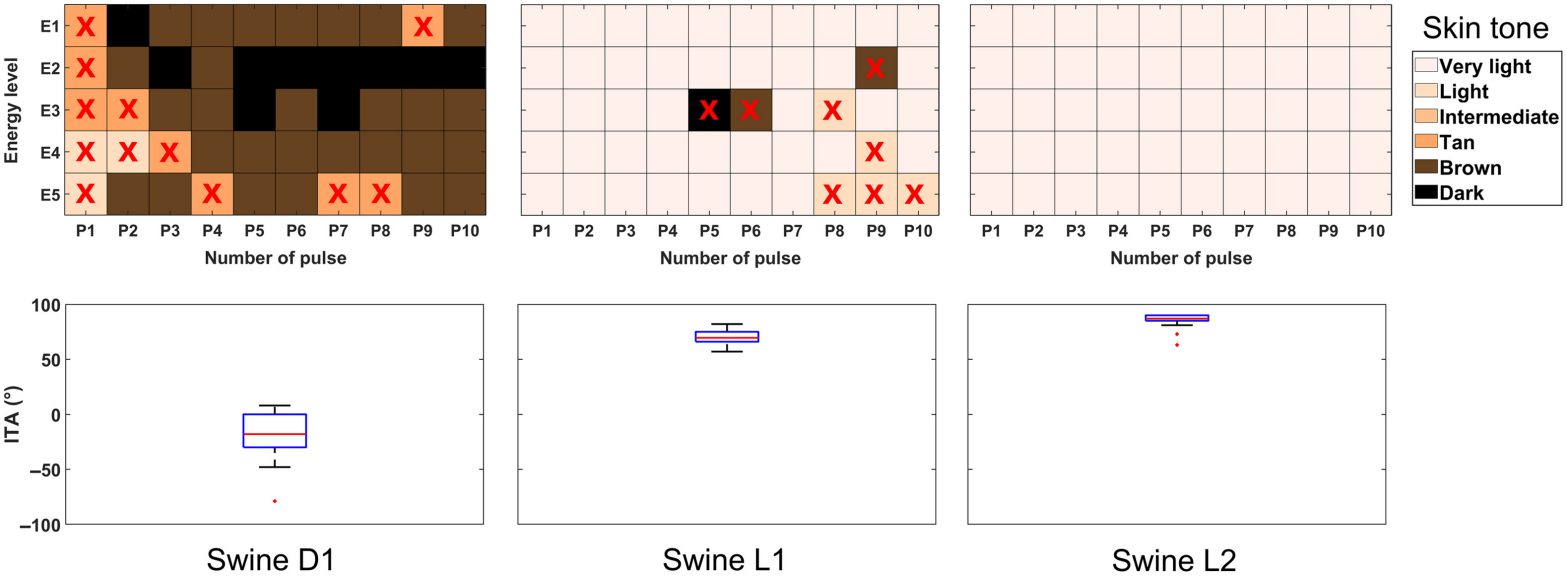
Illustration of discarded skin sites and associated skin-tone classifications of retained skin sites in swine D1, L1, and L2. Regions excluded from analysis are marked with a red "X". The distribution of retained individual typology angle (ITA) values is shown as box-and-whisker plots for each swine. The median ITA is indicated by the red horizontal line, the interquartile range is indicated by the top and bottom bounds of each box, and the maximum and minimum values (excluding outliers, appearing as red datapoints and defined as values >1.5 times the interquartile range) are indicated by the lines extending from each box.

The ITA-based skin-tone categories described in Sec. 2.1 were used to classify local pigmentation at each exposure site. To maximize the contrast in pigmentation and assess potential differences in laser response, only sites in swine L1 and L2 with initial ITA values>55° and sites in swine D1 with initial ITA values≤10° were retained for analysis (e.g., to avoid the areolar region surrounding nipples and more generally to avoid mixing results from light and dark regions in the same group), resulting in 38, 42, and 50 sites for analysis for swine D1, L1, and L2, respectively, as shown in Fig. 2. In particular, skin sites marked with a red “X” were removed, resulting in median ITA values of −18°, 69.5°, and 87° for swine D1, L1, and L2, respectively.

### 2.3 Damage Assessment Process

Each skin region designated for laser exposure was evaluated at four time points: before laser exposure, immediately post-exposure, 5-min post-exposure, and 1-h post-exposure. Photographs were captured at each time point to visually assess the presence or absence of a minimum visible lesion (MVL) resulting from laser exposure. MVL was defined as the earliest observable alteration in skin appearance, characterized by a localized region of erythema distinguishable from the adjacent untreated skin. Three independent observers compared each image with its corresponding baseline (before laser exposure) and determined the presence of erythema, with the final assessment per site established by consensus of at least two of the three independent observers. The proportion of damaged sites was then calculated for each intended exposure level at each post-exposure time point. In addition to visual evaluation, quantitative optical measurements were obtained at each designated region for laser exposure using a colorimeter. In particular, ITA, EI, and MI values were recorded at the same time points as the photographs to quantitatively explain how pigmentation modulates the optical and inflammatory response of the skin after laser exposure.

### 2.4 Quantitative Assessments

#### 2.4.1 ${\mathrm{ED}}_{50}$

In laser safety studies, tissue responses are typically binary and are classified as “lesion” or “no lesion,”^2^ forming the basis for probabilistic dose-response modeling. In this study, the binary outcome was defined as “visible erythema” or “no visible erythema.” Single-pulse exposures were delivered to 10 independent tissue sites at each of five intended laser exposure levels. For each exposure site, the measured radiant exposure dose (i.e., delivered pulse energy divided by beam area) was paired with the corresponding binary erythema response.

Dose–response curves were constructed for swine D1 and L1 by plotting the individual measured radiant exposure values and corresponding binary erythema responses for each exposure site. Probit regression was implemented in MATLAB 2024b (Mathworks, Natick, Massachusetts, United States) using the glmfit function (i.e., generalized linear regression model) with a binomial distribution and probit link, following the standard probit-analysis framework for binary dose–response data.^2^ Radiant exposure values were log-transformed before model fitting. The ${\mathrm{ED}}_{50}$ was calculated as the radiant exposure corresponding to a predicted response probability of 0.5.

To maintain consistency with previous work,^3^^,^^5^ ${\mathrm{ED}}_{16}$ and ${\mathrm{ED}}_{84}$ were additionally calculated and reported, corresponding to exposures producing visible damage in 16% and 84% of sites, respectively. These additional values correspond to tissue response variability and sensitivity around the ${\mathrm{ED}}_{50}$ injury threshold, representing lower and upper bounds around the threshold. The corresponding 95% confidence interval (CI) for each ${\mathrm{ED}}_{50}$ threshold was estimated on the log-exposure scale using the delta method and then back-transformed to the original exposure units.

#### 2.4.2 MI–ITA relationship assessment

To assess the relationship between MI and ITA, a scatter plot of MI versus ITA was generated using data from swine D1, L1, and L2. A second-order polynomial regression analysis was performed to characterize the MI–ITA relationship across the evaluated pigmentation range and to determine the mathematical expression that describes this association. The temporal behavior of this relationship was also assessed at multiple post-exposure time points.

#### 2.4.3 MI–EI relationship assessment

To assess the relationship between MI and EI, a scatter plot of MI versus EI was generated using data from swine D1, L1, and L2. A second-order polynomial regression analysis was performed to characterize the MI–EI relationship across the evaluated pigmentation range and to determine the mathematical expression that describes this association. The temporal behavior of this relationship was also assessed at multiple post-exposure time points.

## 3 Results

### 3.1 Pulse Energy Measurements

**Fig. 3:**
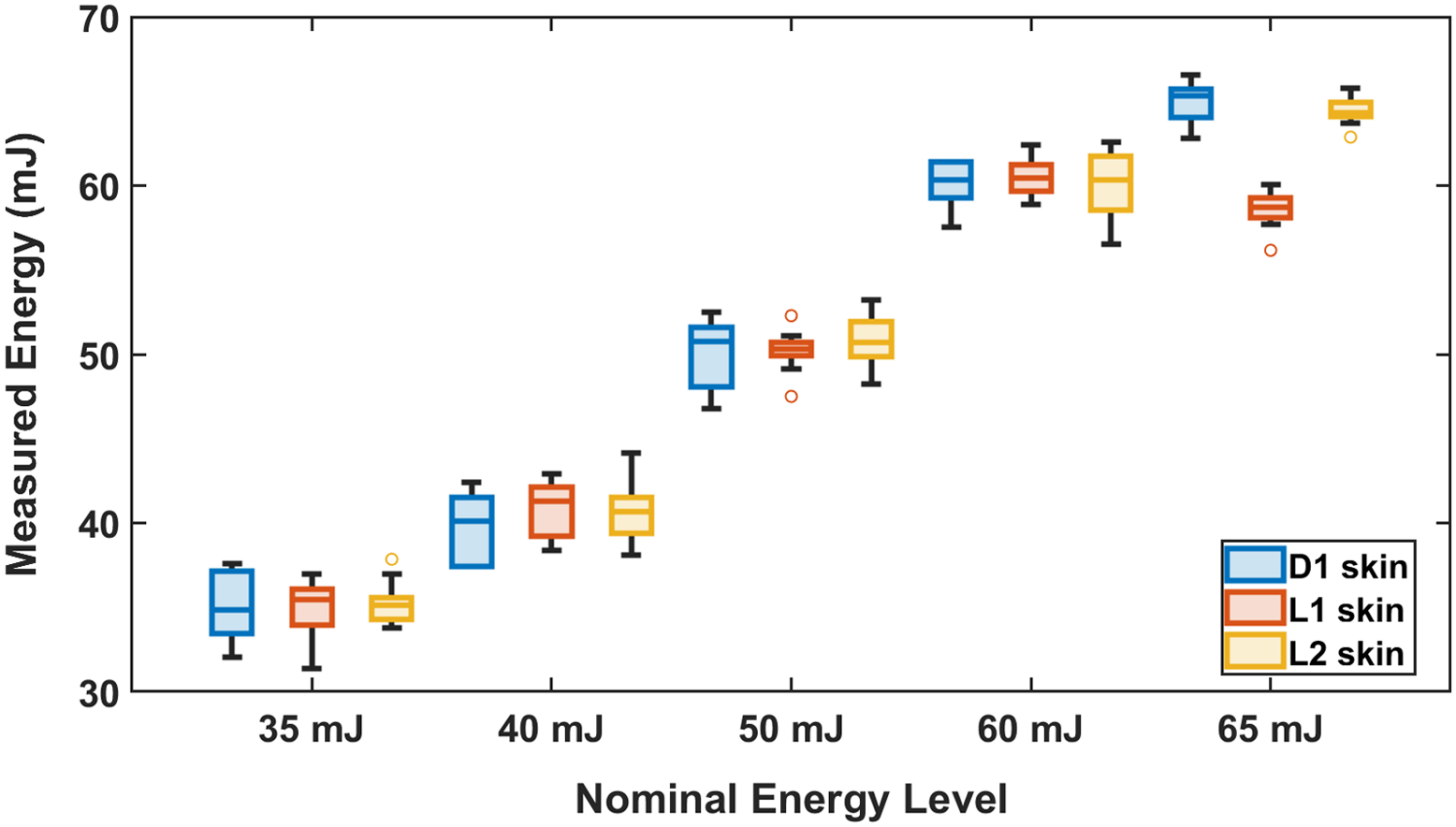
Boxplots of measured pulse energies for five intended laser energies (10 pulses per energy) across the three swine.

Figure 3 shows measured pulse energies for five intended laser energies (i.e., 35, 40, 50, 60, and 65 mJ) across the three swine included in our investigations. The delivered median pulse energies were similar to the intended values, with the exception of the 65 mJ desired energy in swine L1, due to the laser system reaching its maximum achievable energy output during that session. This general consistency enabled the photographs and colorimeter-based optical measurements to be utilized in the ${\mathrm{ED}}_{50}$ injury threshold calculation (with the exception of the 65 mJ desired energy in swine L1, which was omitted from the threshold calculation).

### 3.2 Damage Assessment

**Fig. 4:**
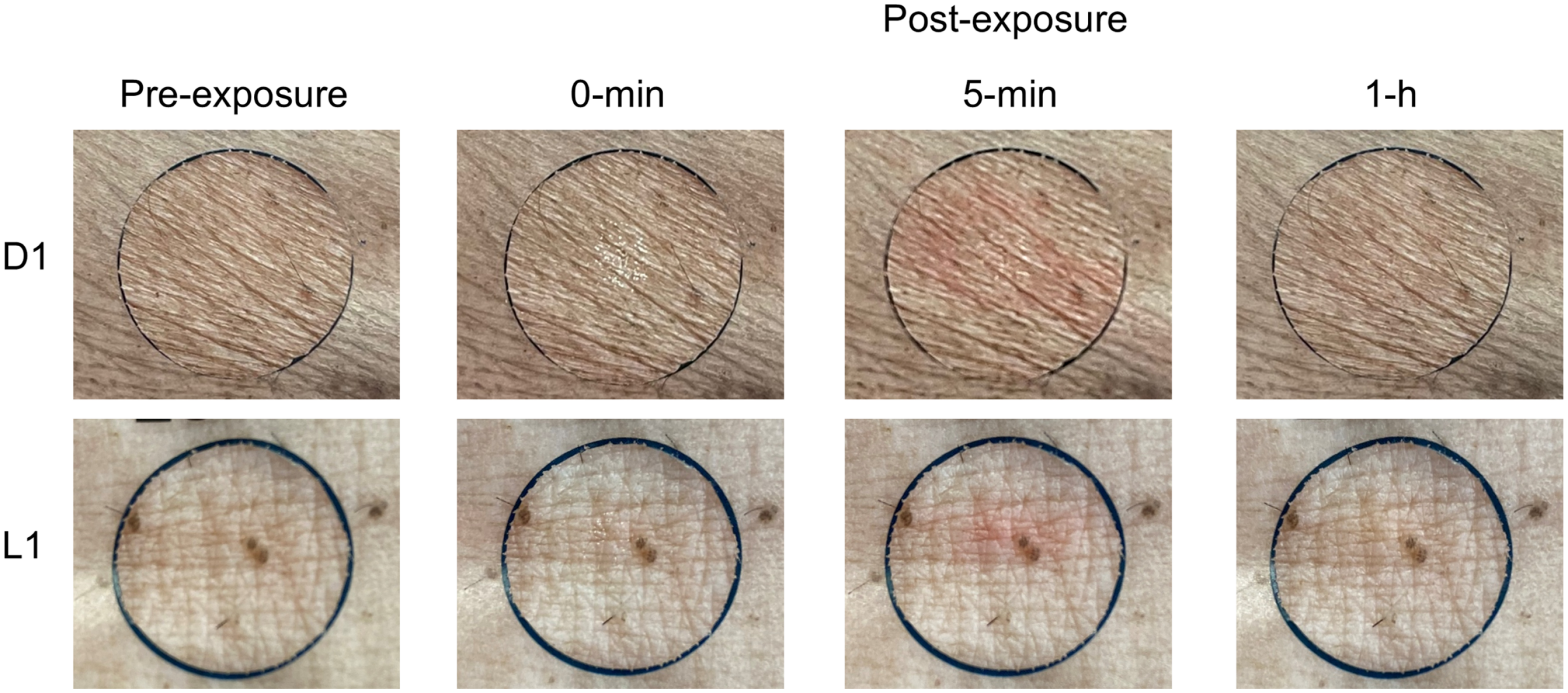
Representative skin sites for swine D1 and L1, showing the presence or absence of blanching and erythema at four time points: pre-exposure, 0-min post-exposure, 5-min post-exposure, and 1-h post-exposure.

Figure 4 shows representative pre- and post-laser exposure photographs illustrating the range of pigmentation responses observed in swine D1 and L1. The images correspond to individual skin sites selected from the grid shown in Fig. 1 (i.e., E3P4 for swine D1 and E3P9 for swine L1). These sites were exposed to single-pulse energies of 49.06 mJ ($\text{radiant}\text{ exposure}=249.86\;\mathrm{mJ}/{\mathrm{cm}}^{2}$) and 51.10 mJ ($\text{radiant}\;\text{exposure}\;=260.25\;\mathrm{mJ}/{\mathrm{cm}}^{2}$), respectively. The pre-exposure ITA values at these locations were −28° (swine D1) and 57° (swine L1). In both swine, blanching was observed immediately following laser exposure (i.e., 0 min post-exposure), appearing as whiter or lighter tissue than the pre-exposure condition. In swine D1, this blanching persisted 5-min post-exposure and remained visible 1-h post-exposure. In swine L1, blanching resolved by the 5-min post-exposure time point. Although recorded as a post-exposure observation, blanching was not used to define a positive visible response for damage assessment. Erythema, which appears as tissue reddening, was visible 5-min post-exposure, with swine D1 showing a greater visible response than swine L1. By 1-h post-exposure, erythema was less pronounced but remained present in a subset of exposure sites. Although probit regression was performed using individual measured radiant exposure/response pairs, the observed response proportions at 0-min-, 5-min-, and 1-h-time points are summarized descriptively in Table 1 according to the intended exposure levels.

**Table 1:** Observed erythema response proportions for swine D1 and L1 at different post-exposure time points and intended energies, which were converted to intended exposures. Variations in denominators reflect differences in the number of evaluated skin sites per exposure level based on pigmentation classification, as described in Sec. 2.2. Data from swine L1 were not available at $331.04\;\mathrm{mJ}/{\mathrm{cm}}^{2}$, as described in Sec. 3.1.

		Observed erythema response proportions based on intended exposures				
		$331.04\;\mathrm{mJ}/{\mathrm{cm}}^{2}$	$305.58\;\mathrm{mJ}/{\mathrm{cm}}^{2}$	$254.65\;\mathrm{mJ}/{\mathrm{cm}}^{2}$	$203.72\;\mathrm{mJ}/{\mathrm{cm}}^{2}$	$178.25\;\mathrm{mJ}/{\mathrm{cm}}^{2}$
D1	0 min	1/8	0/9	0/8	0/7	0/6
	5 min	8/8	9/9	8/8	5/7	5/6
	1 h	3/8	6/9	0/8	1/7	0/6
L1	0 min	—	0/9	0/7	0/9	0/7
	5 min	—	8/9	7/7	4/9	2/7
	1 h	—	1/9	1/7	0/9	0/7

### 3.3 ${\mathrm{ED}}_{50}$

**Fig. 5:**
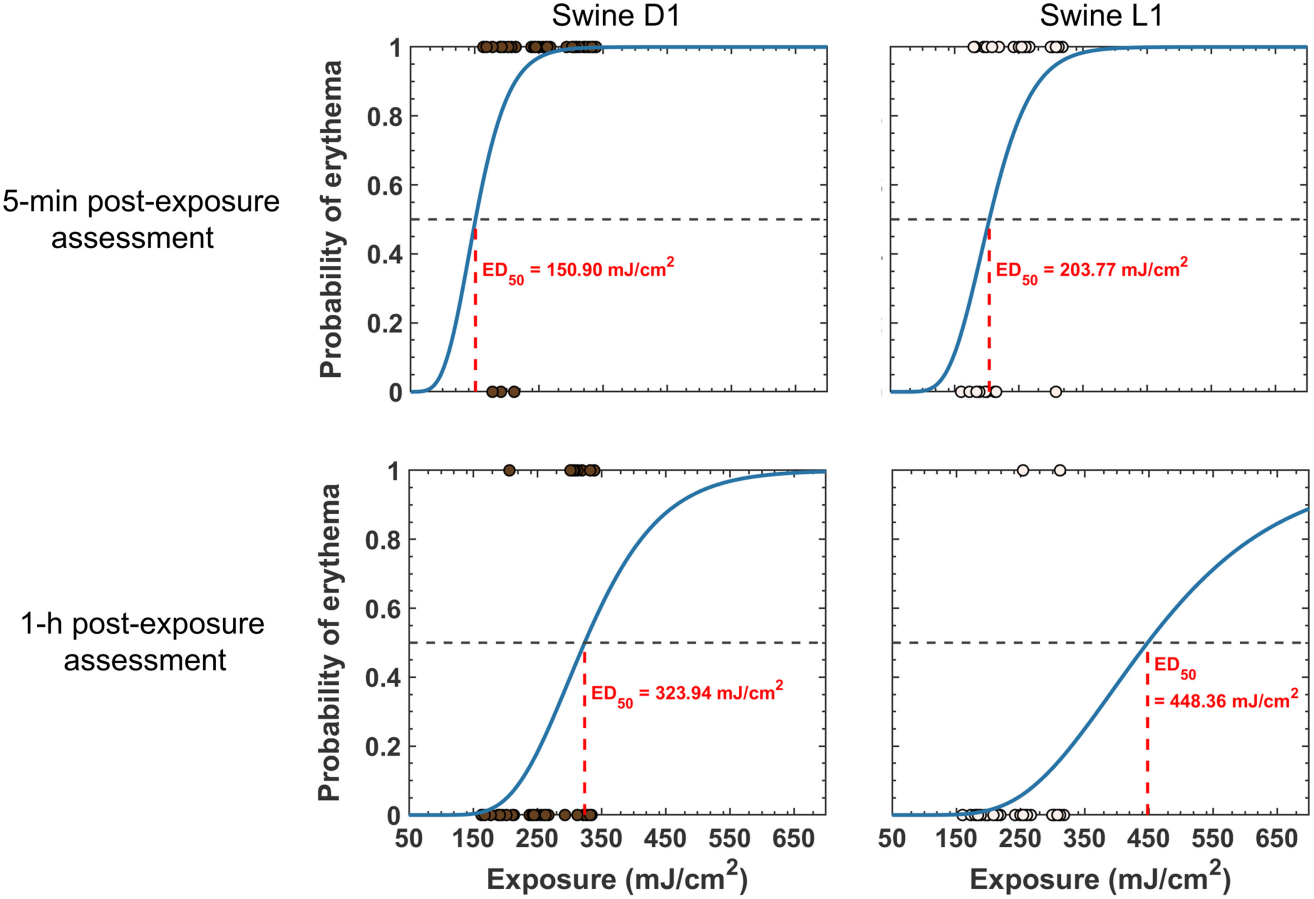
Dose–response curves generated from individual measured radiant exposure values and corresponding binary erythema responses. The blue solid line represents the probit fit obtained using the displayed data points (which represent the measured exposure and probability of erythema pairs).

Figure 5 shows the dose-response curves for swine D1 and L1 based on erythema responses at 5-min and 1-h post-exposure, with corresponding ${\mathrm{ED}}_{16}$, ${\mathrm{ED}}_{50}$, ${\mathrm{ED}}_{84}$, and ${\mathrm{ED}}_{50}$ confidence intervals summarized in Table 2. The 1-h erythema endpoint was used as the primary endpoint for ${\mathrm{ED}}_{50}$ calculation. The 5-min erythema assessment is provided as a descriptive early-response measure. Based on the 1-h endpoint, swine D1 has an injury threshold that is 27.7% lower than that of swine L1. Although there is overlap among the confidence intervals reported in Table 2, there is a trend toward higher exposure levels required to produce a persistent visible erythema response in lighter skin. The wider confidence interval for swine L1 reflects the limited number of positive responses at the 1-h assessment endpoint.

**Table 2:** Dose–response parameters derived from probit analysis of erythema at 1-h and 5-min post-exposure in swine D1 and L1.

	5-min assessment		1-h assessment	
	Swine D1	Swine L1	Swine D1	Swine L1
${\mathrm{ED}}_{16}(\mathrm{mJ}/{\mathrm{cm}}^{2})$	115.52	158.46	243.79	311.51
${\mathrm{ED}}_{50}(\mathrm{mJ}/{\mathrm{cm}}^{2})$	150.90	203.77	323.94	448.36
${\mathrm{ED}}_{50}\;95\%\;\mathrm{CI}(\mathrm{mJ}/{\mathrm{cm}}^{2})$	103.91 to 219.15	177.48 to 233.94	272.63 to 384.92	190.74 to 1,054.00
${\mathrm{ED}}_{84}(\mathrm{mJ}/{\mathrm{cm}}^{2})$	197.54	261.96	429.90	645.30
${\mathrm{ED}}_{50}$ (mJ, 5-mm-diameter fiber)	29.63	40.01	63.61	88.04

### 3.4 MI–ITA Relationship

**Fig. 6:**
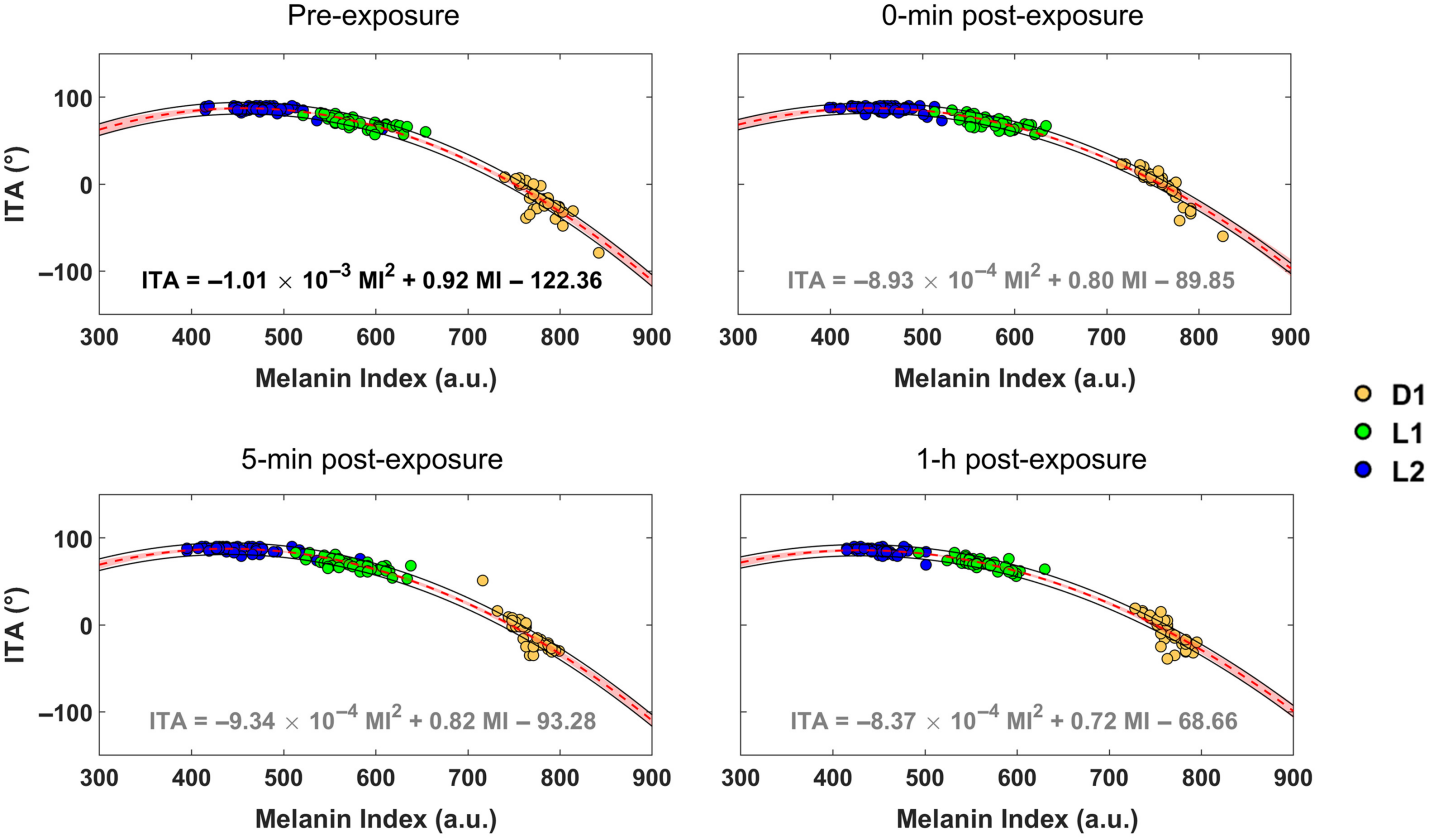
Relationship between ITA and MI for three swine pre- and post-laser exposure. Colored markers represent individual colorimeter measurements. Red dashed line shows corresponding quadratic polynomial fit to data points in each plot. The transparent red band represents the 95% confidence interval (CI) of the fit, indicating the uncertainty of the polynomial regression. The black solid lines above and below the fit represent ± one standard deviation of the residuals (which represents the variability of ITA values relative to the fit).

Figure 6 shows ITA as a function of MI for multiple time points pre- and post-exposure. Each curve was fit using a second-degree polynomial, with $R^{2}$ values ranging from 0.97 to 0.98, which is considered an excellent fit. Therefore, any of the coefficients can be used to describe the fit. However, given that post-exposure changes such as blanching may alter the colorimeter readings, the representative MI–ITA relationship was only derived from pre-exposure data to represent a baseline skin condition in the absence of laser-induced optical changes. The resulting quadratic equation and associated coefficients (shown in black in Fig. 6) are:

$$\mathrm{ITA}=-1.01\times 10^{-3}\cdot {\mathrm{MI}}^{2}+0.92\cdot \mathrm{MI}-122.36.$$ (2)

### 3.5 MI–EI Relationship

**Fig. 7:**
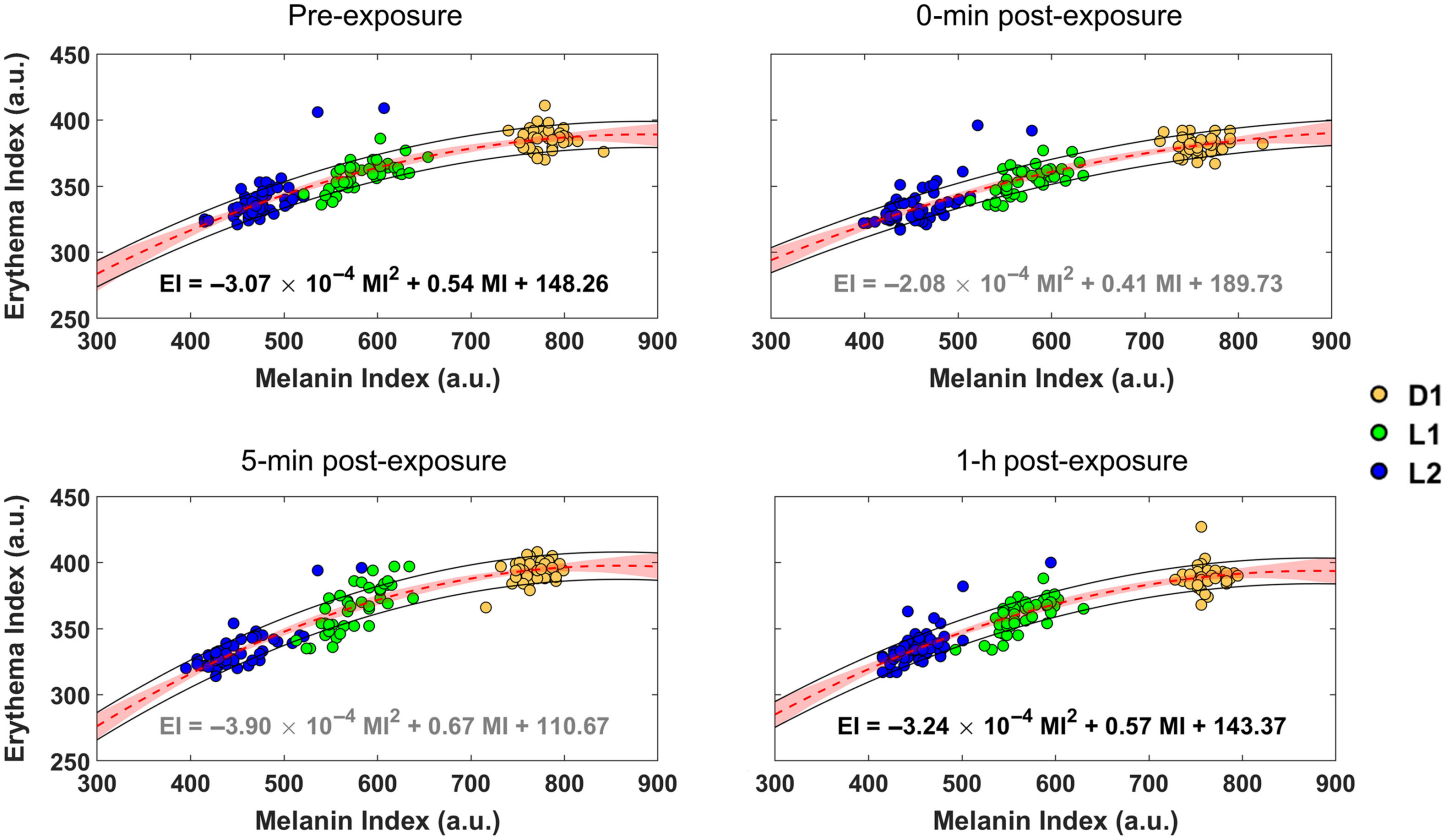
Relationship between EI and MI for three swine pre- and post-laser exposure. Colored markers represent individual colorimeter measurements. Red dashed line shows corresponding quadratic polynomial fit to data points in each plot. The transparent red band represents the 95% confidence interval (CI) of the fit, indicating the uncertainty of the polynomial regression. The black solid lines above and below the fit represent ± one standard deviation of the residuals (which represents the variability of EI values relative to the fit).

Figure 7 shows EI as a function of MI for multiple time points pre- and post-exposure, which were each fit to a second-degree polynomial. The resulting curves were highly consistent across time, with $R^{2}$ values ranging from 0.81 to 0.87, demonstrating the fit robustness. Based on these results, two representative polynomial equations were derived. The first equation describes EI as a function of MI in the absence of visible erythema (baseline) and was obtained from the polynomial coefficients shown in the pre-exposure panel of Fig. 7:

$${\mathrm{EI}}_{\text{baseline}}=-3.07\times 10^{-4}\cdot {\mathrm{MI}}^{2}+0.54\cdot \mathrm{MI}+148.26.$$ (3)

The second equation describes EI as a function of MI once erythema was present and was derived from the polynomial coefficients shown in the 1-h post-exposure panel of Fig. 7:

$${\mathrm{EI}}_{\text{erythema}}=-3.24\times 10^{-4}\cdot {\mathrm{MI}}^{2}+0.57\cdot \mathrm{MI}+143.37.$$ (4)

## 4 Discussion

We present the first known systematic experimental quantification of pigmentation-dependent laser injury thresholds in swine skin at 750-nm-wavelength using nanosecond laser pulses. Swine D1 (i.e., dark-skinned swine with median abdominal ITA value of −18°) had 27.7% lower ${\mathrm{ED}}_{50}$ injury threshold than swine L1 (i.e., light-skinned swine with median abdominal ITA value of 69.5°), which is consistent with increased epidermal melanin absorption reported on more pigmented skin. Similar pigmentation-dependent disparities have been reported in dermatology, where laser parameters derived primarily from light-skinned cohorts are associated with increased adverse responses in darker skin.^29^^,^^30^ By directly measuring injury thresholds across pigmentation phenotypes, these results provide experimental context for understanding how biological variability influences tolerance to nanosecond laser exposure at 750-nm-wavelength. These findings are particularly relevant for optics-based imaging modalities such as photoacoustic imaging, in which allowable optical fluence directly governs signal-to-noise ratio, penetration depth, and diagnostic performance.

The lower ${\mathrm{ED}}_{50}$ measured in swine D1 relative to swine L1 was consistent across the 5-min and 1-h endpoints (Table 2). Therefore, under the evaluated 750-nm nanosecond-pulse conditions, darker skin is susceptible to developing visible erythema with less radiant exposure than that required for lighter skin. This observation differs from the photoprotective role of melanin under ultraviolet (UV) irradiation (i.e., 100 to 400 nm wavelength), where melanin attenuates incident radiation and reduces UV-induced DNA damage.^31^ This difference reflects the distinct roles of melanin across the two exposure regimes. Under UV irradiation, melanin attenuates incident radiation and protects cellular targets. However, at a wavelength of 750 nm, melanin absorption can increase local energy deposition and contribute to the visible tissue response. More generally, optical tissue injury depends on wavelength, pulse duration, and irradiation conditions and may result from photochemical, photothermal, or photomechanical processes.^32^ From this perspective, swine L2 did not develop visible erythema within the tested exposure range (as noted in Sec. 2.1) likely because of the higher ITA values (Fig. 6), which indicate lower melanin content. Considering that melanin is a dominant absorber at 750-nm wavelength,^6^ the lower melanin content in the skin of swine L2 is expected to reduce optical absorption and local energy deposition. This reduced energy deposition is assumed to be responsible for the absence of visible erythema, suggesting that the injury threshold for swine L2 exceeded the exposures used in this study.

Beyond injury thresholds and the erythema response differences observed across swine D1, L1, and L2, the quantitative relationship derived between ITA and MI provides a continuous description of baseline skin color and pigmentation across the evaluated skin-tone range (Fig. 6). Although ITA is widely used as a device-independent descriptor of skin tone^17^ and strongly correlates with melanin concentration,^33^ it is typically used as a categorical classifier rather than as a continuous variable. By deriving a polynomial relationship that quantitatively relates ITA to MI across the full range of possible ITA values (i.e., between −90° and 90°), this work demonstrates that ITA can also be used as a continuous predictor of pigmentation rather than solely as a classification metric.

The ability of colorimeter-derived metrics (i.e., ITA, EI, and MI) to explain binary erythema outcomes at matched exposure levels was limited. Mixed positive and negative erythema responses were observed at the same exposure levels (Fig. 5). However, the relationships among ITA values, MI values, and EI changes between post-exposure and pre-exposure measurements at specific, individual exposure sites (i.e., ΔEI) were inconsistent with the binary erythema classifications, as shown in the Appendix. Therefore, ITA, MI, EI, and ΔEI should not be interpreted as direct surrogates for binary visible-response assessment in this dataset. This lack of one-to-one correspondence may be attributed to measurement variability, local tissue heterogeneity, and visual features not captured by EI alone.

Prior laser-skin-injury studies have defined ${\mathrm{ED}}_{50}$ thresholds using lesion assessment at 24 to 48 h post-exposure. At wavelengths of 1318, 1214, and 2000 nm, Winston et al.,^34^ Chen et al.,^35^ and Chen et al.,^3^ respectively, used 24 to 48 h endpoints for millisecond-to-second pulse durations. Similarly, DeLisi et al.^36^ employed a 24 to 48 h endpoint at 1064 nm despite using nanosecond pulses. These longer evaluation windows help distinguish persistent tissue injury from transient visible responses. However, 1-h assessments have also been reported in swine skin laser damage threshold studies,^37^^,^^38^ demonstrating minimal differences between 1- and 24-h thresholds. Herein, the 1-h erythema endpoint was therefore used as the primary endpoint for ${\mathrm{ED}}_{50}$ calculation, as it represented the more persistent visible response within our study constraints, with the 5-min erythema response representing a descriptive early-response measure. Although this early response may be relevant for understanding acute visible tissue changes or device-use considerations (e.g., pain or sensitivity thresholds), it should not be interpreted as equivalent to a conventional persistent tissue injury.

At a wavelength of 750 nm, ANSI Z136.1 specifies a single MPE of $25.2\;\mathrm{mJ}/{\mathrm{cm}}^{2}$ for skin.^1^ This study reports pigmentation-specific ${\mathrm{ED}}_{50}$ thresholds. While the safety margin is not explicitly defined for our study parameters, generally, MPEs have been approximated by applying an order of magnitude reduction as a safety margin to the experimentally determined ${\mathrm{ED}}_{50}$ threshold.^3^ Based on this convention, when we compare the ANSI MPE with the ${\mathrm{ED}}_{50}$ thresholds derived from the primary 1-h erythema endpoint in our study, the resulting safety margins are 12.9 (${\mathrm{ED}}_{50}=323.94\;\mathrm{mJ}/{\mathrm{cm}}^{2}$) for dark skin (swine D1), and 17.8 (${\mathrm{ED}}_{50}=448.36\;\mathrm{mJ}/{\mathrm{cm}}^{2}$) for light skin (swine L1). Applying the same calculation to the secondary 5-min erythema endpoint yields safety margins of 6.0 (${\mathrm{ED}}_{50}=150.90\;\mathrm{mJ}/{\mathrm{cm}}^{2}$) for dark skin (swine D1), and 8.1 (${\mathrm{ED}}_{50}=203.77\;\mathrm{mJ}/{\mathrm{cm}}^{2}$) for light skin (swine L1). Therefore, two different skin tones can provide two different safety margins for the same experimental parameters. These observations highlight the importance of experimentally quantifying how injury thresholds vary with pigmentation to better contextualize the safety margins implicit in existing exposure guidelines, which is particularly relevant for systems designed to operate near regulatory limits.

One limitation of this study is that the 5-min erythema responses were weighted toward positive outcomes, whereas the 1-h erythema responses included fewer positive outcomes (Fig. 5), which increased uncertainty in the probit-derived ${\mathrm{ED}}_{50}$ estimates and confidence intervals reported in Table 2. In addition, the ${\mathrm{ED}}_{50}$ threshold for swine L1 (i.e., $448.36\;\mathrm{mJ}/{\mathrm{cm}}^{2}$) was estimated by extrapolation beyond the highest analyzed exposure (i.e., $305.58\;\mathrm{mJ}/{\mathrm{cm}}^{2}$), contributing to its wide confidence interval. Therefore, future studies would benefit from pilot testing or a wider available pulse-energy range to better sample both the low-response and high-response regions of the dose–response curve. Another consideration is the potential contribution of pigmented hair follicles to local optical absorption. Pigmented hair follicles can act as localized absorbers and may contribute to thermal hot spots that influence tissue response assessment and corresponding injury thresholds at melanin-absorbing wavelengths. In this study, shaving enabled the removal of surface hair to improve optical coupling while preserving the follicular structure of skin, thereby maintaining native skin architecture. Laser-induced erythema was not visibly localized around hair follicles during post-exposure assessment. However, follicular melanin may have contributed to local thermal heterogeneity and therefore to the measured ${\mathrm{ED}}_{50}$ values. Hair follicles may therefore represent an additional source of variability, contributing to differences in site-level colorimeter-derived measurements.

Future work will focus on developing a simulation framework that can be parameterized by skin tone, enabling predictive modeling of nanosecond laser–tissue interactions, using the pigmentation-dependent relationships established in this study. This framework will allow the generation of skin-specific optical profiles and, ultimately, the prediction of pigmentation-dependent injury thresholds across the continuum of skin pigmentation.

## 5 Conclusion

The work presented herein provides the first known experimental pigmentation-specific ${\mathrm{ED}}_{50}$ exposure thresholds for nanosecond illumination at 750-nm wavelength. A 27.7% lower injury threshold was observed in dark-skinned swine (median ITA=−18°, ${\mathrm{ED}}_{50}=323.94\;\mathrm{mJ}/{\mathrm{cm}}^{2}$) compared with light-skinned swine (median ITA=69.5°, ${\mathrm{ED}}_{50}=448.36\;\mathrm{mJ}/{\mathrm{cm}}^{2}$), demonstrating that differences in epidermal melanin content translate into measurable differences in exposure tolerance at wavelengths where melanin is a primary optical absorber. Therefore, darker skin has greater susceptibility to damage with the investigated laser parameters, relative to lighter skin. These results provide experimental context for interpreting the safety margins associated with uniform MPE limits. In addition, the derived mathematical relationship linking ITA to MI establishes a quantitative description of baseline skin color and pigmentation. Together, these measurements and model provide a quantitative experimental basis to understand the pigmentation-dependent variability in laser–tissue interactions and its relevance to the safe design and evaluation of optics-based technologies.

## Appendix

**Fig. 8:**
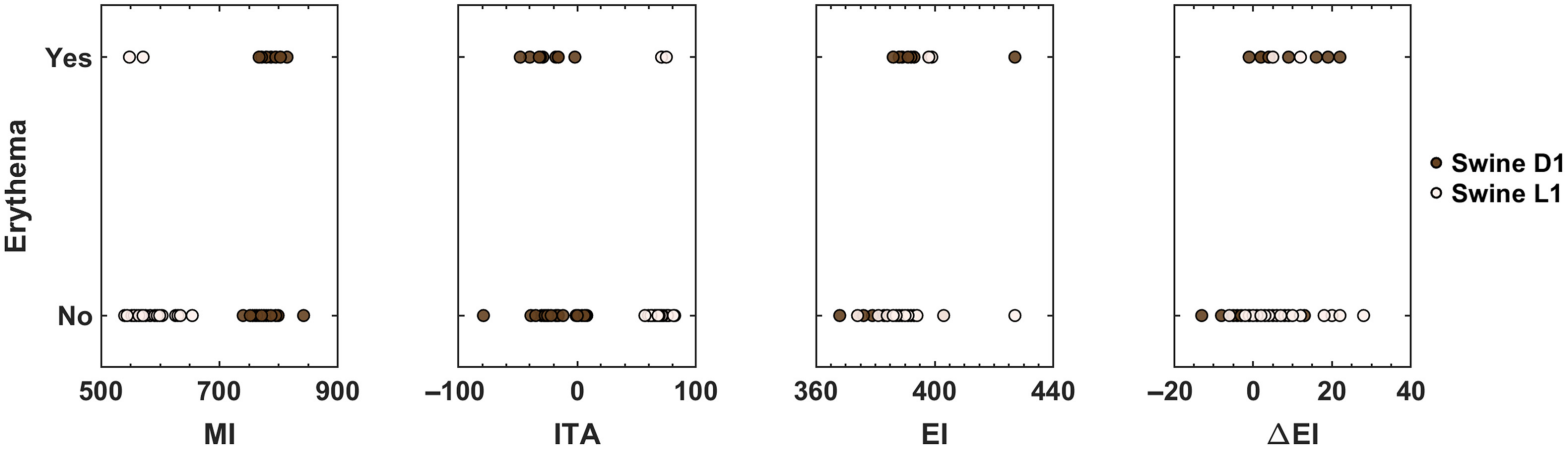
1-h erythema response for swine D1 and L1 plotted as functions of MI, ITA, EI, and ΔEI.

To assess whether mixed responses at identical pulse energies were associated with local colorimeter measurements, the 1-h erythema responses were plotted as functions of MI, ITA, EI, and ΔEI (i.e., EI changes between post-exposure and pre-exposure measurements at a specific exposure site). As shown in Fig. 8, these variables did not yield a reliable separation between sites with and without erythema. Instead, similar overlap was observed in each case. These plots demonstrate that the erythema responses are not a function of MI, ITA, EI, or ΔEI.

Although a similar overlap is observed in Figs. 8 and 5, these two results have different interpretations. In Fig. 5, mixed erythema outcomes at similar radiant exposures are expected in the transition region of the dose–response curve and provide the variability needed to estimate ${\mathrm{ED}}_{50}$, which corresponds to a 50% erythema probability. Outside of this transition region, the erythema response is more predictably expected to be positive when the exposures are higher (or negative when the exposures are lower). However, in Fig. 8, the overlap demonstrates that the colorimeter measurements are not directly correlated with the presence or absence of erythema at each individual skin site.

## Data Availability

Code, data, and materials used in this study are proprietary to the PULSE Lab and can be made available upon reasonable request to the PULSE Lab director (MALB). Fulfillment of such requests requires involvement and signed agreements between users and the relevant office(s) at Johns Hopkins University.
